# Altered localisation of the copper efflux transporters ATP7A and ATP7B associated with cisplatin resistance in human ovarian carcinoma cells

**DOI:** 10.1186/1471-2407-8-175

**Published:** 2008-06-19

**Authors:** Ganna V Kalayda, Christina H Wagner, Irina Buß, Jan Reedijk, Ulrich Jaehde

**Affiliations:** 1 Department of Clinical Pharmacy, Institute of Pharmacy, University of Bonn, An der Immenburg 4, 53121 Bonn, Germany; 2 Leiden Institute of Chemistry, Leiden University, P.O. Box 9502, 2300 RA Leiden, The Netherlands

## Abstract

**Background:**

Copper homeostasis proteins ATP7A and ATP7B are assumed to be involved in the intracellular transport of cisplatin. The aim of the present study was to assess the relevance of sub cellular localisation of these transporters for acquired cisplatin resistance *in vitro*. For this purpose, localisation of ATP7A and ATP7B in A2780 human ovarian carcinoma cells and their cisplatin-resistant variant, A2780cis, was investigated.

**Methods:**

Sub cellular localisation of ATP7A and ATP7B in sensitive and resistant cells was investigated using confocal fluorescence microscopy after immunohistochemical staining. Co-localisation experiments with a cisplatin analogue modified with a carboxyfluorescein-diacetate residue were performed. Cytotoxicity of the fluorescent cisplatin analogue in A2780 and A2780cis cells was determined using an MTT-based assay. The significance of differences was analysed using Student's *t *test or Mann-Whitney test as appropriate, p values of < 0.05 were considered significant.

**Results:**

In the sensitive cells, both transporters are mainly localised in the *trans-*Golgi network, whereas they are sequestrated in more peripherally located vesicles in the resistant cells. Altered localisation of ATP7A and ATP7B in A2780cis cells is likely to be a consequence of major abnormalities in intracellular protein trafficking related to a reduced lysosomal compartment in this cell line. Changes in sub cellular localisation of ATP7A and ATP7B may facilitate sequestration of cisplatin in the vesicular structures of A2780cis cells, which may prevent drug binding to genomic DNA and thereby contribute to cisplatin resistance.

**Conclusion:**

Our results indicate that alterations in sub cellular localisation of transport proteins may contribute to cisplatin resistance *in vitro*. Investigation of intracellular protein localisation in primary tumour cell cultures and tumour tissues may help to develop markers of clinically relevant cisplatin resistance. Detection of resistant tumours in patients may in turn enable individualization of the chemotherapy in the early stage of treatment.

## Background

Despite the success of cisplatin-based anticancer chemotherapy, its application is limited because in most cases, tumours develop resistance after repeated administrations of the drug. Acquired cisplatin resistance is a net result of multiple pathways, which often act simultaneously in a given cell [1,2]. Furthermore, primary mechanisms that determine a resistant phenotype vary significantly between different cancer cell lines [3], and also defects in drug transport appear to play a major role in development of resistance [4,5]. The mechanisms mediating uptake, intracellular transport and efflux of cisplatin remain largely obscure [6]. In the past decade, a number of studies have provided evidence that the copper homeostasis proteins ATP7A and ATP7B are involved [7-10]. These P-type ATPases have been suggested to either sequester cisplatin in vesicular structures or to mediate efflux of the drug [11,12]. Increased expression of ATP7A and ATP7B has been associated with acquired resistance to cisplatin in cancer cell line models and in clinical samples [7,8,11,13,14].

Under basal conditions, when extracellular copper concentrations are low, ATP7A and ATP7B are localised in the *trans-*Golgi network. Exposure to increased copper levels results in re-localisation of ATP7A to the plasma membrane and of ATP7B to intracellular vesicular compartments. For both proteins, this re-localisation is reversible once copper has been removed from the culture medium. Copper-regulated trafficking of ATP7A and ATP7B is believed to be important for maintaining copper homeostasis [15-17]. The effect of cisplatin on sub cellular localisation of these copper transporters was studied in cell lines molecularly engineered to express either ATP7A or ATP7B. In these cell lines, which exhibited a biologically relevant degree of resistance, both transporters were found to localise in the *trans-*Golgi network in the absence of cisplatin. Cisplatin exposure triggered re-localisation of ATP7B (and not ATP7A) from the *trans-*Golgi to more peripherally located vesicles [10,18,19]. These findings suggest that ATP7B mediates cisplatin efflux, while ATP7A is rather involved in the intracellular sequestration of the drug [12].

The issue of sub cellular localisation of ATP7A and ATP7B in cell lines, selected for cisplatin resistance, has not yet been addressed. Recently, we characterised an A2780/A2780cis cisplatin-sensitive/resistant human ovarian carcinoma cell line pair with respect to cisplatin sensitivity, drug accumulation and efflux, DNA platination, as well as expression of copper homeostasis proteins [20]. In the present study, we have investigated localisation of ATP7A and ATP7B in these cell lines and found that both proteins have a different localisation pattern in the resistant cells as compared to the sensitive cells. Here we discuss the factors that likely account for changes in sub cellular localisation of ATP7A and ATP7B and consider relevance of altered protein localisation for decreased cisplatin sensitivity in the resistant cell line.

## Methods

### Materials

Bafilomycin A_1_, MTT (MTT = 3-(4,5-dimethylthiazol-2-yl)-2,5-diphenyl-2H-tetrazolium bromide), Triton X-100, bovine serum albumin (BSA), ribonuclease A (RNAse A), fluorescein isothiocyanate-dextran (FITC-dextran, M = 70000), GelMount™ mounting medium and cis-diamminedichloridoplatinum(II) (cisplatin) were obtained from Sigma (Steinheim, Germany). LysoTracker™ Red DND-99, Hoechst 33342, ALEXA Fluor™ 488- and ALEXA Fluor™ 594-conjugated chicken anti-goat antibodies, NBD-C_6_-ceramide (6-((N-(7-nitrobenz-2-oxa-1,3-diazol-4-yl)amino)hexanoyl)-sphingosine) complexed to BSA, propidium iodide were ordered form Invitrogen (Karlsruhe, Germany). Goat antibodies to ATP7A and ATP7B were from Santa Cruz Biotechnology, Inc. (Santa Cruz, CA, USA). All solvents were reagent grade and were used as purchased. [{1-([5-(and-6)-carboxyfluorescein diacetate]aminomethyl)-1,2-ethylenediamine}dichloridoplatinum(II)] (CFDA-Pt) was synthesised according to the previously published method [21]. Briefly, the protection Boc (t-butyloxycarbonyl) group of [{1-(t-butyloxycarbonyl)-aminomethyl)-1,2-ethylenediamine}dichloridoplatinum(II)] was removed in 0.1 M HCl at 60°C overnight. The resulting solution was neutralised to pH 7.5 with 2 M NaOH and allowed to react with 0.9 eq 5-(and-6)-carboxyfluorescein diacetate, succinimidyl ester (Invitrogen, Karlsruhe, Germany), for 15 min at 0°C and subsequently for 30 min at room temperature in order to obtain the desired compound. The resulting precipitate was centrifuged off and successively washed with water, ethanol end ether. The product was characterised by ^1^H and ^195^Pt NMR spectroscopy and mass spectrometry.

### Cell lines and growth conditions

A2780 and A2780cis (cisplatin-resistant) human ovarian carcinoma cell lines were grown as monolayers in RPMI-1640 medium supplemented with 10% foetal calf serum, L-glutamine, penicillin and streptomycin (Sigma, Steinheim, Germany) in a humidified 5% CO_2_, 95% air atmosphere.

### Drug sensitivity in the A2780 and A2780cis cell lines

Cytotoxicity of cisplatin, CFDA-Pt and bafilomycin A_1 _in A2780 and A2780cis cells was evaluated using an MTT-based assay [22]. After trypsinisation, the cells were divided in 96-well plates at concentrations of 10^4 ^cells/well in 100 μl growth medium. The cells were allowed to attach overnight. Then, medium was removed and stock solutions of cisplatin in millipore water (2 mg/ml), CFDA-Pt in dimethylformamide (50 mM) and bafilomycin A_1 _in dimethylsulfoxide (100 mM) were diluted in medium. Six subsequent dilutions each were added to the cells in quadruplicate (100 μl/well). Some control wells contained 0.5% dimethylformamide, which corresponded to the highest solvent concentration in the dilutions of CFDA-Pt. After 72 h of incubation, 50 μl of a 5 mg/ml MTT solution in phosphate buffered saline (PBS) was added to each well, and the cells were incubated at 37°C for about 90 min. Subsequently, medium was discarded and 100 μl of dimethylsulfoxide was added to each well, yielding purple solutions. The optical density was measured at 590 nm using a Multiskan™ microplate reader (ThermoLabsystems, Dreieich, Germany). The results were analysed and the pEC_50 _values (pEC_50 _= -log EC_50_, EC_50 _is the drug concentration that produces 50% of the maximum possible response) were determined with the GraphPad Prism™ analysis software package (GraphPad Software, San Diego, USA) using non-linear regression (sigmoidal dose response, variable slope).

### Lysosomal and endosomal acidification

Acidification of lysosomal and endosomal compartments in A2780 and A2780cis cells was compared using FITC-dextran. Labelling of endosomes and lysosomes with FITC-dextran was performed according to the literature procedures [23,24]. Briefly, A2780 and A2780cis cells were divided in a 96-well plate at concentrations of 10^5 ^cells/well in 100 μl growth medium. The cells were allowed to attach for 6 h. Subsequently, the medium was discarded and FITC-dextran (5 mg/ml) dissolved in serum-free medium was added to the cells. To label endosomes the cells were incubated with FITC-dextran for 5 min, to label lysosomes they were incubated with FITC-dextran for 30 min followed by washing with serum-free medium and subsequent incubation with this medium for 90 min. After labelling of endosomes or lysosomes, the cells were washed with serum-free medium, and complete medium was added to the cells. Fluorescence intensity was measured using a Polarstar-Galaxy™ microtitre plate reader (BMG-Lab-Technologies, Offenburg, Germany).

### Flow cytometry

10^6 ^cells (A2780 or A2780cis) were incubated with LysoTracker™ Red DND-99 (1 μM) in RPMI-1640 for 30 min, centrifuged, washed with the medium, and re-suspended in normal growth medium. Then fluorescence was measured using a FACSort™ flow cytometer (BD Biosciences).

### Immunohistochemical staining

Immunohistochemical staining was done one day after seeding cells on cover slips. In some experiments, cells were pre-treated with either 5 μM cisplatin, 5 μM CFDA-Pt or 200 nM bafilomycin A_1 _for 1 h. Lysosomes were labelled in RPMI-1640 containing 1 μg/ml LysoTracker™ Red DND-99 at 37°C for 1 h. In some experiments, nuclei were stained with 5 μM Hoechst 33342 at 37°C for 1 h. After three rinses with PBS, cells were fixed with 3.7% formaldehyde in PBS for 15 min at room temperature. After fixation, cells were washed three times with PBS and permeablilised with 0.5% Triton X-100 in PBS for 30 min. Labelling of the *trans-*Golgi network was performed with 5 μM NBD-C_6_-ceramide complexed to BSA at 37°C for 30 min. Cells were then rinsed with PBS, blocked for 1 h in PBS containing 1% BSA followed by incubation for 90 min at 37°C with a primary antibody against the respective transport protein (ATP7A or ATP7B). Subsequently, cells were washed with PBS and incubated for 90 min at 37°C with the secondary ALEXA Fluor™ 488- or ALEXA Fluor™ 594-conjugated antibody. Antibody solutions were diluted in PBS containing 1% BSA. To stain the nucleus with propidium iodide in some samples, cells were treated with RNAse A (100 μg/ml in PBS) for 30 min at 37°C, washed with PBS and incubated with 5 μM propidium iodide in PBS for 15 min at 37°C. After the final washing steps, the cells were gradually dehydrated in an ethanol series of 70%, 90%, 100%, for 1 min each. The cover slips were mounted in GelMount™ medium for microscopic observations. Images showing staining of lysosomes with LysoTracker™ Red in both cell types are representative each of five images, which were recorded using an Olympus U-TBI90 confocal system at Laboratory of Molecular Immunology, LIMES Institute, University of Bonn. Other images in this article are each representative of five images taken using a Leica TCS SP2 confocal system at Laboratory for Molecular Developmental Biology, LIMES Institute, University of Bonn. Each fluorochrome was scanned individually and each image included 5 – 20 cells. The number of cells showing a certain pattern (e.g. TGN localised protein) was counted in every image and expressed as a percentage of all cells in the image. Then the median percentage values from different images were calculated.

### Statistics

The significance of differences was analysed using Student's *t *test or Mann-Whitney test as appropriate, p values of <0.05 were considered significant.

## Results

### Sub cellular localisation of ATP7A and ATP7B

**Figure 1:**
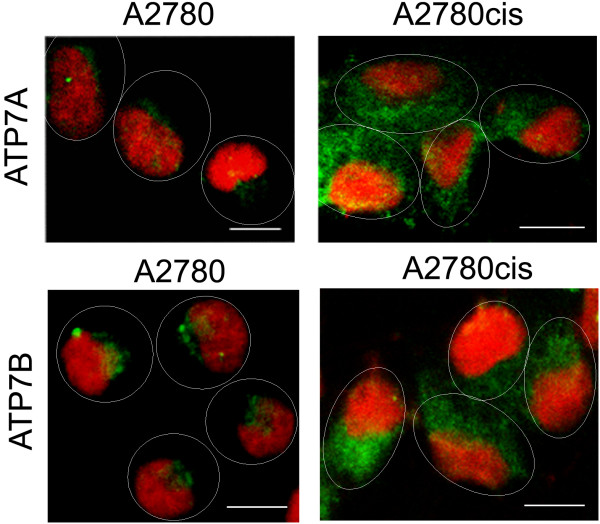
**Sub cellular localisation of ATP7A and ATP7B in A2780 and A2780cis cells**. Immunofluorescence localisation of ATP7A and ATP7B (both *green*) in A2780 and A2780cis cells. Cell nuclei were stained with propidium iodide (*red*). Ovals indicate cell periphery. Scale bar, 10 μm.

**Figure 2:**
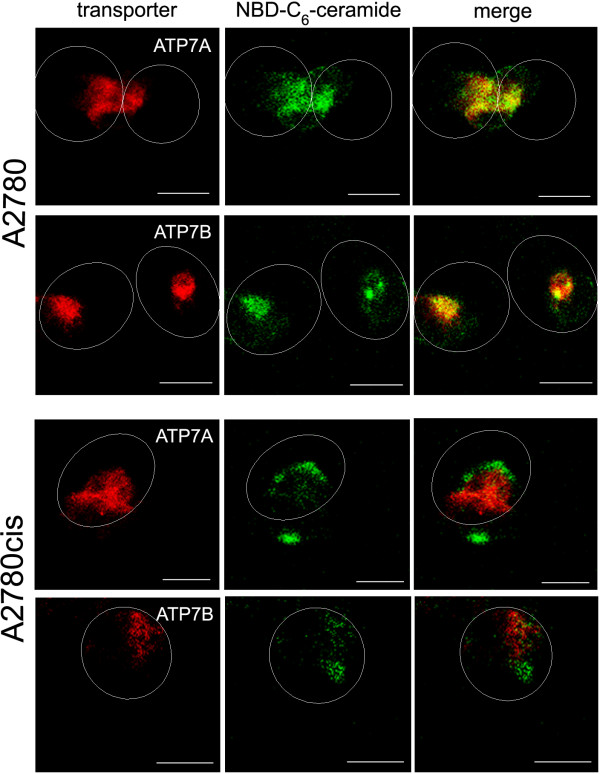
**Co-localisation of ATP7A and ATP7B with a *trans-*Golgi network marker**. Co-localisation of markers for ATP7A and ATP7B (both r*ed*) with NBD-C_6_-ceramide (*green*), a marker for the *trans-*Golgi network. *Yellow*, the structure is positive for Golgi and protein markers. Ovals indicate cell periphery. Scale bar, 10 μm.

In A2780 cell line, the fluorescent signals corresponding to ATP7A and ATP7B are limited to the perinuclear regions of the cells (Figure 1). Previously published studies in other cell systems reported localisation of both proteins in the *trans-*Golgi network [16-19,25,26]. However, in cisplatin-resistant A2780cis cells the proteins are distributed away from the perinuclear region to more peripherally located vesicles in the cytosol (Figure 1). The percentage of sensitive and resistant cells showing dispersed localisation of the proteins is given in Table 1. We also distinguished between dividing (located next to each other) and non-dividing cells since dividing cells are more likely to show dispersed protein localisation. In order to confirm localisation of ATP7A and ATP7B in the *trans-*Golgi network of the sensitive cells, and re-localisation of the transporters in the resistant cells, co-localisation experiments using NBD-C_6_-ceramide, a fluorescent marker for the Golgi complex, were performed. Images presented in Figure 2 show substantial co-localisation of both proteins with NBD-C_6_-ceramide in A2780 cells and a negligible degree of co-localisation in A2780cis cells. The percentage of cisplatin-sensitive and -resistant cells that show co-localisation of the proteins with the Golgi marker is presented in Table 2.

**Table 1:** Median percentage of A2780 and A2780cis cells showing dispersed localisation of ATP7A and ATP7B.

	Cell line	All cells	p	Dividing cells	p (div. cells)	Non-dividing cells	p (non-div. cells)
	A2780	16.2		20.0		0	
ATP7A	A2780cis	88.8	0.0286	82.6	0.0286	100.0	0.0286

	A2780	11.1		12.5		10	
ATP7B	A2780cis	80.2	0.0286	82.5	0.0286	80	0.0286

Median percentage values were calculated from five individual images. The significance of differences was analysed using Mann-Whitney test.

**Table 2:** Median percentage of A2780 and A2780cis cells showing co-localisation of ATP7A and ATP7B with the Golgi marker.

	A2780	A2780cis	p
ATP7A	92.8	0.0	0.0159
ATP7B	88.9	15.4	0.0079

Median percentage values were calculated from five individual images. The significance of differences was analysed using Mann-Whitney test.

**Figure 3:**
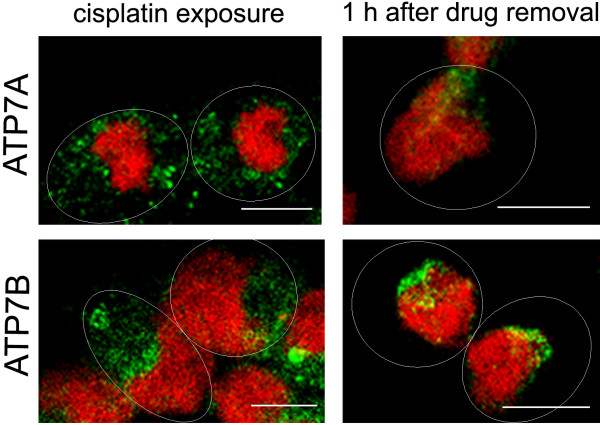
**Localisation of ATP7A and ATP7B in A2780 cells after cisplatin exposure**. Immunofluorescence localisation of ATP7A and ATP7B (both *green*) in A2780 cells after cisplatin exposure for 1 h and subsequent incubation of the cells in the drug-free medium for 1 h. Cell nuclei were stained with propidium iodide (*red*). Ovals indicate cell periphery. Scale bar, 10 μm.

In order to investigate the effect of drug exposure on protein localisation, A2780 cells were treated with 5 μM cisplatin for 1 h. Cisplatin triggered redistribution of ATP7A and ATP7B from the perinuclear region to more peripherally located sites in the cytosol. Interestingly, localisation of both proteins in the area close to the nucleus was fully restored within 1 h after removal of the drug from the culture medium (Figure 3). The percentage of cells, which show dispersed localisation of the transporters in A2780 cells after cisplatin treatment and after subsequent incubation with the drug-free medium was determined, and the values are presented in Table 3. However, since no inhibitors of protein synthesis were used, return of ATP7A and ATP7B to localisation in the *trans-*Golgi network may or may not reflect a retrograde traffic of the proteins.

**Table 3:** Median percentage of A2780 cells showing dispersed localisation of ATP7A and ATP7B after cisplatin exposure and after subsequent incubation with the drug-free medium.

	Cisplatin exposure	1 h after drug removal	p
ATP7A	76.5	10.5	0.0043
ATP7B	70.8	12.2	0.0043

Median percentage values were calculated from five individual images. The significance of differences was analysed using Mann-Whitney test.

Rapid cisplatin-induced re-localisation of ATP7A and ATP7B towards the cell periphery and quick return of the proteins back to the *trans-*Golgi network suggests that cisplatin-controlled trafficking of the transporters may indicate an efflux pathway of the drug. Cisplatin binding to the protein may result in protein redistribution to (secretory) vesicles, followed by drug excretion and re-localisation of the protein back to the *trans-*Golgi network. Cisplatin exposure had no effect on protein localisation in the resistant cell line (images not shown).

### Constitutive recycling of ATP7A and ATP7B in A2780 cells

**Figure 4:**
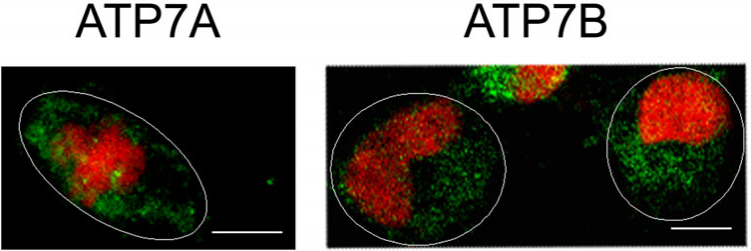
**Effect of bafilomycin A_1 _on localisation of ATP7A and ATP7B**. Immunofluorescence localisation of ATP7A and ATP7B (both *green*) in A2780 cells treated with 200 nM bafilomycin A_1 _for 1 h. Cell nuclei were stained with propidium iodide (*red*). Ovals indicate cell periphery. Scale bar, 10 μm.

Some cellular proteins (e.g. furin and TGN38) are known to continuously recycle between the *trans-*Golgi network and the plasma membrane and to be localised in the *trans-*Golgi by a process of continuous retrieval from the cell surface [27,28]. Drugs which inhibit endosomal recycling lead to sequestration of these proteins in vesicles of the endosomal pathway [29,30]. In order to investigate, whether ATP7A and ATP7B continuously recycle between the *trans-*Golgi network and the cell periphery, the effect of bafilomycin A_1_, a drug that blocks endosomal retrieval by inhibiting the vacuolar H^+^-ATPase [31], on intracellular localisation of ATP7A and ATP7B in the sensitive A2780 cells was studied. As shown in Figure 4, exposure of A2780 cells to 200 nM bafilomycin A_1 _for 1 h triggered redistribution of the proteins from the *trans-*Golgi to more peripherally located vesicular structures. This was the case in 77.5% cells for ATP7A and in 72.7% cells for ATP7B (median percentage is given). These results provide evidence of continuous recycling of ATP7A and ATP7B between the Golgi apparatus and cell periphery in the sensitive cells. Therefore, in the sensitive cell line the proteins are likely to localise in the *trans-*Golgi network by a process of continuous retrieval from the peripherally located vesicles.

In order to investigate whether arrest of endosomal recycling can render A2780 cells resistant to cisplatin, we intended to assess the influence of bafilomycin A_1 _on cisplatin sensitivity. Unfortunately, it was not possible because of the adverse effect of bafilomycin A_1 _treatment on cell viability. The EC_50 _values for bafilomycin A_1 _were determined as 8.5 nM (pEC_50 _= 8.07 ± 0.14, mean ± SE) in the A2780 cell line and 12 nM (pEC_50 _= 7.92 ± 0.1, mean ± SE) in the A2780cis cell line. However, the concentrations of the drug, which do not affect cell viability (<10 nM), are not high enough to block the activity of a vacuolar H^+^-ATPase [32].

### Comparison of lysosomal and endosomal acidification in A2780 and A2780cis cells

**Figure 5:**
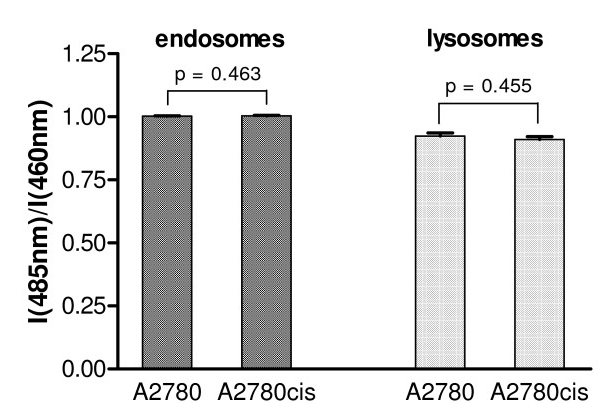
**Comparison of lysosomal and endosomal acidification**. Comparison of lysosomal and endosomal acidification in A2780 and A2780cis cells using ratios of emission intensities at 520 nm at the two excitation wavelengths (485 nm and 460 nm) after FITC-dextran labelling of endosomes and lysosomes, respectively. The results are expressed as means ± SE of emission intensity ratios in 24 wells (endosomes) and 48 wells (lysosomes) from two independent experiments. The significance of differences was analysed using Student's *t *test.

Similar protein localisation patterns in A2780 cells after incubation with H^+^-ATPase inhibitor bafilomycin A_1 _and in A2780cis cells suggest that defects in lysosomal/endosomal acidification in the A2780cis cell line may be responsible for different localisation of ATP7A and ATP7B. We used FITC-dextran to compare acidification of endosomes and lysosomes in the sensitive and resistant cells. The emission intensity of FITC at 520 nm increases with increasing pH at λ_ex _= 485 nm but it is unaffected by pH at λ_ex _= 460 nm. Thus, the ratio of emission intensities at the two excitation wavelengths provides information about acidification of a given cellular compartment, which is independent on FITC concentration [24]. Early endosomes were labelled with FITC-dextran in cell culture medium for 5 min as described previously [23], and lysosomes were labelled with FITC-dextran for 30 min followed by 90 min incubation with the label-free medium following the literature procedure [24]. The ratios of FITC emission intensities after excitation at 485 nm and 460 nm are presented in Figure 5. As is clear from Figure 5, no notable difference in acidification of either endosomes or lysosomes between A2780 and A2780cis cells was observed.

### Reduced lysosomal compartment in cisplatin-resistant cells

**Figure 6:**
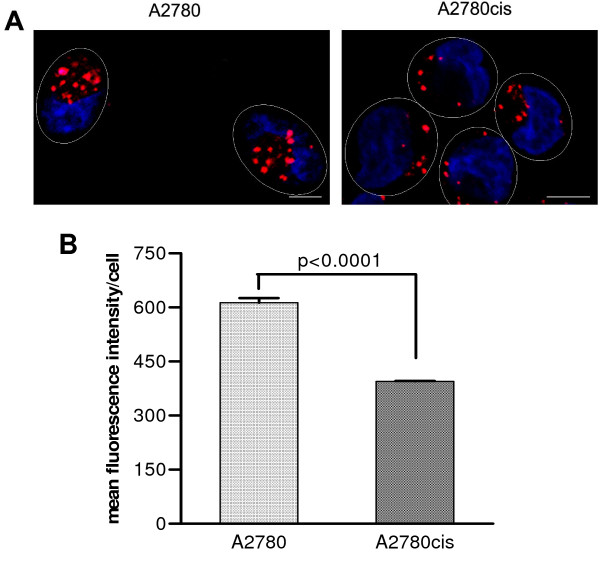
**Lysosomal compartments in A2780 and A2780cis cells**. Comparison of the lysosomal compartments in A2780 and A2780cis cells using confocal microscopy (A) and flow cytometry (B). A. Lysosomes were labelled with LysoTracker™ Red (*red*), cell nuclei were stained with Hoechst 33342 (*blue*). Ovals indicate cell periphery. Scale bar, 5 μm. B. The results represent means ± SE of four experiments. The significance of differences was analysed using Student's *t *test.

The lysosomal dye LysoTracker™ Red DND-99 was used to compare lysosomal compartments in the A2780 and A2780cis cell lines. This lysosomal dye has been chosen for its ability to be retained in acidic organelles after formaldehyde fixation. Images of sensitive and resistant cells in which lysosomes were stained with LysoTracker™ Red and nuclei were labelled with Hoechst 33342 are presented in Figure 6A and show that cisplatin-resistant cells contain notably less acidic vesicles compared to their sensitive counterparts. This observation was confirmed by a flow cytometry study. Average fluorescence intensity of the lysosomal dye per cell was significantly lower in the resistant cell line indicating a reduced lysosomal compartment (Figure 6B).

### Co-localisation of ATP7A and ATP7B with a fluorescent cisplatin analogue

**Figure 7:**
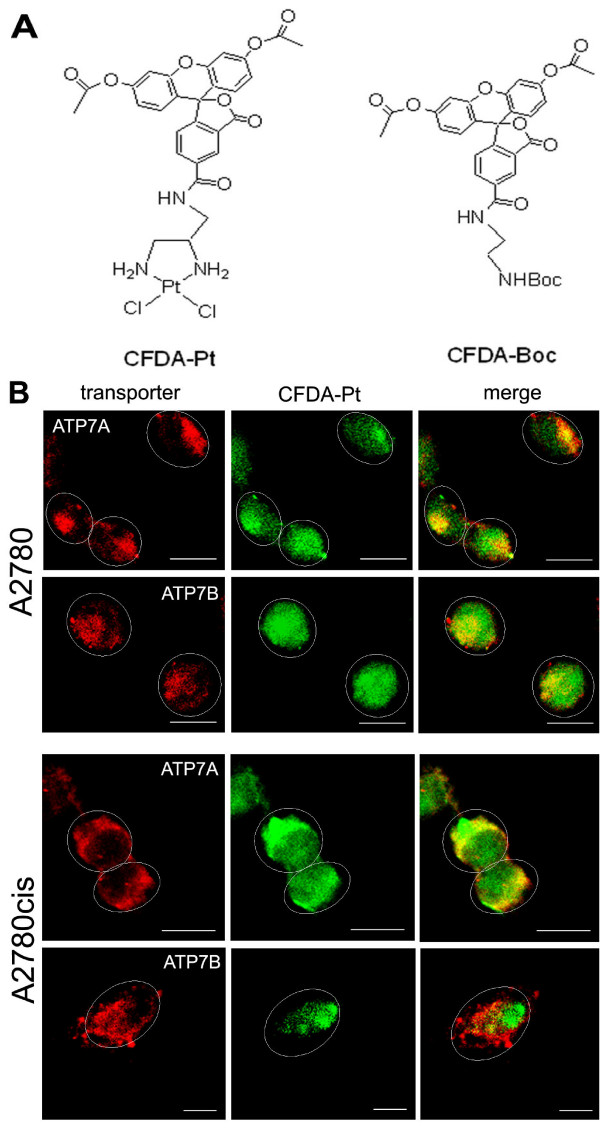
**Co-localisation of ATP7A and ATP7B with CFDA-Pt**. A. Structure of the fluorescent cisplatin analogue (CFDA-Pt) and the platinum-free fluorescein derivative (CFDA-Boc). B. Co-localisation of CFDA-Pt (*green*) and markers for ATP7A and ATP7B (both *red*) in A2780 and A2780cis cells. *Yellow*, the structure is positive for CFDA-Pt and protein markers. Ovals indicate cell periphery. Scale bar, 10 μm.

In order to reveal whether ATP7A and ATP7B mediate cisplatin transport in A2780 and A2780cis cells, co-localisation experiments using a fluorescent cisplatin analogue labelled with carboxyfluorescein-diacetate (CFDA-Pt, Figure 7A) were performed. Earlier studies reported that accumulation of CFDA-Pt is lower in cisplatin-resistant cells in parallel to decreased cisplatin uptake in these cells as compared to their sensitive counterparts [33]. In addition, the cellular distribution pattern of CFDA-Pt was found to be significantly different to that of the platinum-free 1-([5-(and-6)-carboxyfluorescein diacetate]-2-(*tert*-butyloxycarbonyl)-1,2-ethylenediamine (CFDA-Boc, chemical structure is depicted in Figure 7A), indicating that the intracellular trafficking of CFDA-Pt is determined by the platinum moiety and not by the fluorescent label [21]. These results suggested that CFDA-Pt represents a suitable model compound for investigation of the cellular processing of cisplatin. However, antitumor activity of this complex had not been investigated, so in order to validate suitability of CFDA-Pt for our cellular system, we compared cytotoxicity of CFDA-Pt and cisplatin in A2780 and A2780cis cells. As presented in Table 4, CFDA-Pt was found to be less cytotoxic than cisplatin in both cell lines. Nevertheless, it exhibited substantial activity in A2780 cells and its activity was significantly reduced in the A2780cis cell line indicating that cisplatin-resistant cells are also resistant to CFDA-Pt (~4-fold resistance). These data provide evidence that CFDA-Pt can be used as a model complex in fluorescence microscopy investigations of intracellular distribution of cisplatin.

**Table 4:** Sensitivity of A2780 and A2780cis cells to cisplatin and CFDA-Pt.

	A2780		A2780cis		
			
	pEC_50_	EC_50_, μM	pEC_50_	EC_50_, μM	p
cisplatin	5.455 ± 0.0941	3.5	4.631 ± 0.0095	23.4	0.0001
CFDA-Pt	4.445 ± 0.1524	35.9	3.856 ± 0.0711	139.3	0.0127

Sensitivity of A2780 and A2780cis cells to cisplatin and CFDA-Pt was assessed using an MTT-based assay. The pEC_50 _values are means ± SE of four experiments. The significance of differences was analysed using Student's *t *test.

Images of A2780 and A2780cis cells incubated with CFDA-Pt and subsequently stained with antibodies to ATP7A and ATP7B are presented in Figure 7B. In the sensitive cells, both proteins showed extensive co-localisation with CFDA-Pt. In the resistant cells, however, only ATP7A, and not ATP7B, co-localised with the fluorescent cisplatin analogue. Thus, both proteins are likely to mediate transport of CFDA-Pt in the A2780 cells but only ATP7A seems to be involved in transport of the drug in A2780cis cells. Interestingly, after the treatment with CFDA-Pt both ATP7A and ATP7B showed dispersed localisation in A2780 cells and this may indicate that CFDA-Pt induces re-localisation of the transporters to the cell periphery in the sensitive cells in the same way as cisplatin does. However, images in Figure7B do not present sufficient proof of protein re-localisation following exposure to CFDA-Pt.

## Discussion

### Sub cellular localisation of ATP7A and ATP7B in sensitive and resistant cells

In previous work from our laboratory, we assessed the role of copper homeostasis proteins CTR1, ATP7A and ATP7B in uptake and efflux of cisplatin in A2780 human ovarian carcinoma cell line and its cisplatin-resistant variant A2780cis cell line as well as the relevance of these transporters for tumour cell sensitivity to cisplatin [20]. Intracellular accumulation of cisplatin was found to be markedly reduced in the resistant A2780cis cell line, which is likely to result (al least in part) from the decreased expression of CTR1, a copper influx transporter believed to mediate cisplatin uptake. However, no difference in the rate of cisplatin efflux between sensitive and resistant cell line was observed, although resistant cells overexpressed the efflux transporter ATP7A. Expression of ATP7B, another transport protein previously reported to be involved in cisplatin efflux, was comparable in sensitive and resistant cells. This finding correlated well with similar efflux rates in both cell lines, however, it did not agree with the results of other groups, which linked increased expression of ATP7B to cisplatin resistance in different clonal cell lines [10,11].

In an attempt to understand these contradictions, we examined the sub cellular localisation of ATP7A and ATP7B in A2780 sensitive and A2780cis resistant cells. Our results show that both proteins have different localisation patterns in A2780 and A2780cis cells. In the sensitive cells, both transporters localise in the *trans-*Golgi network, which is consistent with the previously reported observations in a number of other cell lines [16-19,25,26]. However, in the resistant cells, ATP7A and ATP7B are distributed away from the *trans-*Golgi network to more peripherally located vesicles in the cytosol. It is not clear, to which cellular compartments these vesicles belong. It is not likely that ATP7A and ATP7B are re-localised to the lysosomal compartment given that the latter is significantly reduced in A2780cis cells. Both ATP7A and ATP7B have been previously suggested to accumulate cisplatin in vesicles of the secretory pathway [10,18,34]. However, the possibility that the transporters are localised in vesicles of the endosomal compartment can not be ruled out. In any event, it should be pointed out that vesicular structures expressing ATP7A in the A2780cis cell line do not express ATP7B and vice versa, since ATP7A-containing vesicles co-localise with a fluorescent cisplatin analogue CFDA-Pt while vesicles containing ATP7B do not.

In A2780 cells, both transporters appear to undergo constitutive recycling between the *trans-*Golgi network and more peripherally located vesicles in the cytosol. Cisplatin exposure triggers rapid trafficking of ATP7A and ATP7B from the Golgi complex towards the cell periphery. The observed shift in steady-state distribution of the proteins is reversible upon removal of cisplatin from the culture medium. This system of cisplatin-regulated trafficking of ATP7A and ATP7B may indicate how cisplatin efflux by these transporters is achieved. In fact, both proteins have been reported to undergo copper-regulated trafficking between the *trans-*Golgi network and the plasma membrane (ATP7A) or vesicular structures at the cell periphery (ATP7B) as a way to maintain copper homeostasis [15-17]. Copper-induced trafficking of ATP7A and ATP7B was also observed in A2780 cells (images not shown). However, in our study it was not possible to distinguish between the sites of ATP7A and ATP7B re-localisation after treatment with copper or cisplatin. Thus, our results suggest that A2780 cells utilise the same vesicular export pathway for efflux of cisplatin as they employ for export of copper.

### Possible causes of altered localisation of transporters in resistant cells

Continuous recycling of the copper efflux transporters between the Golgi complex and peripherally located vesicles in the cytosol appears to be blocked in the cisplatin-resistant A2780cis cell line. As a result, the proteins are sequestrated in vesicular structures in the cytosol. Looking for possible cellular alterations, which may account for different localisation of ATP7A and ATP7B in the resistant cells, we compared acidification of the lysosomal and endosomal compartment in A2780 and A2780cis cell lines. Vesicular trafficking and protein turnover are very sensitive to even small changes in organelle pH and we hypothesised that altered localisation of ATP7A and ATP7B in the resistant cells may be a consequence of acidification defects of the lysosomal and/or endosomal compartment. Some cisplatin-resistant cell lines have previously been reported to have less acidic lysosomes than their sensitive counterparts [35-38]. However, no notable differences in acidification of either lysosomes or endosomes between sensitive and resistant cells were observed in our case.

Interestingly, the cisplatin-resistant A2780cis cells were found to have significantly less lysosomes than the sensitive A2780 cells. Lysosomes play an important role in the vesicular trafficking [39]. They represent a terminal degradative compartment of the endocytic pathway. Lysosomes receive their enzymes from the *trans-*Golgi network mainly *via *late endosomes (also called multivesicular bodies), which also sort internalised proteins for recycling or degradation in lysosomes [40]. Unfortunately, the mechanism of delivery of endocytosed material from endosomes to lysosomes is not well elucidated [41]. Several possible pathways have been proposed [41] including maturation of late endosomes into lysosomes, vesicular transport from late endosomes to lysosomes, so-called 'kiss-and-run' (a cycle of transient contacts between endosomes and lysosomes, which allow exchange of the material between them, followed by dissociation) and direct fusion to form a hybrid organelle, which contains both late endosome and lysosome components and where the endocytic cargo is either recycled or digested. Any of these mechanisms represent a sequence of highly regulated intracellular events and even small alterations may lead to the failure of the whole pathway to function properly. A reduction of the lysosomal compartment in the cisplatin-resistant cell line may indicate decreased protein trafficking from late endosomes to lysosomes due to mis-sorting of proteins in late endosomes and may reflect a general defect in intracellular protein trafficking, which may prevent constitutive recycling of ATP7A and ATP7B and eventually lead to sequestration of these transporters in the vesicular structures of resistant cells. Our results confirm findings of other groups, that the lysosomal compartment in cells with acquired resistance to cisplatin features major abnormalities, which eventually results in changes in protein processing [35-37,42]. We suggest that the reduced lysosomal compartment may reflect defects in protein recycling, which may in turn be responsible for altered localisation of the efflux transporters ATP7A and ATP7B in the A2780cis resistant cell line.

### Consequences of altered localisation of the transporters in resistant cells

In order to investigate possible consequences of altered localisation of ATP7A and ATP7B, co-localisation experiments using a fluorescent cisplatin analogue modified with carboxyfluorescein-diacetate (CFDA-Pt) were carried out. Some reports in the literature [6,43] argue that CFDA-Pt is not likely to behave similarly to cisplatin inside the cell because a large lipophilic fluorophore would significantly change the pharmacokinetic properties of the complex. However, there are several lines of evidence that CFDA-Pt represents a suitable model complex for investigation of intracellular transport of cisplatin. Firstly, the cellular distribution of CFDA-Pt was found different from that of the platinum-free fluorophore CFDA-Boc [21,33]. Secondly, cellular accumulation of CFDA-Pt in cisplatin-resistant U2-OS/Pt osteosarcoma cells was found to be reduced compared to its accumulation in sensitive U2-OS cells [33], which is in agreement with decreased cisplatin accumulation in the U2-OS/Pt cell line [33,44]. Finally, the results of the cytotoxicity tests presented here show that CFDA-Pt exhibits substantial activity in the A2780 cell line and cross-resistance with cisplatin in the A2780cis cell line. Taken together, these findings strongly suggest that modification of cisplatin with carboxyfluorescein-diacetate does not alter the pharmacological properties of the complex, which are important for the cisplatin-resistant phenotype.

Co-localisation experiments showed that CFDA-Pt is associated with ATP7A and ATP7B in cisplatin-sensitive A2780 cells. Both transporters have been previously reported to co-localise with a fluorescein-labelled cisplatin analogue in vesicles of the secretory pathway [18,34]. Based on the data obtained using different transfected cell lines it has been suggested that ATP7B mediates cisplatin efflux, whereas ATP7A functions to sequester the drug in the vesicular compartment [12]. On the contrary, in the A2780 cell line both proteins appear to be involved in cisplatin efflux, since cisplatin triggers trafficking of both transporters away from the *trans-*Golgi network to vesicular compartments on the cell periphery. It should, however, be pointed out that the cell lines transfected with ATP7A and ATP7B exhibited biologically relevant degree of cisplatin resistance and cannot, therefore, be directly compared with cisplatin-sensitive cell lines. Otherwise, since cisplatin serves as a substrate for both ATP7A and ATP7B, it is also possible that the presence of considerable amounts of both transporters in the cell in some way influences their function, which is not the case in a clonal cell line engineered to overexpress one of the proteins.

In the cisplatin-resistant cell line, ATP7A was found to co-localise with CFDA-Pt, and only a negligible degree of co-localisation between the complex and ATP7B was observed. Therefore, in spite of altered localisation ATP7A appears to mediate either efflux or sequestration of cisplatin in the A2780cis cells. Sequestration is more likely to take place given higher expression of the transporter in the resistant cell line and nonetheless similar efflux rate in both cell lines [20]. It is also in agreement with previously reported data, which suggested that ATP7A regulates cell sensitivity to cisplatin by sequestrating it in the vesicular compartment [11,12]. In contrast, re-localisation of ATP7B away from the *trans-*Golgi network to peripherally located vesicles appears to prevent cisplatin binding to this protein and does not allow ATP7B to perform cisplatin efflux in the resistant cells. As a result, the drug becomes sequestrated intracellularly, possibly in ATP7A-expressing vesicles.

In the sensitive A2780 cells, both ATP7A and ATP7B are likely to mediate cisplatin transport. Due to altered localisation of the proteins in the resistant A2780cis cell line, ATP7A appears to dominate over ATP7B in cisplatin binding and to determine the intracellular fate of the drug. This is in agreement with increased expression of ATP7A and unchanged expression levels of ATP7B in resistant cells compared to their sensitive counterparts [20]. Given peripheral localisation of ATP7A in A2780cis cells, cisplatin may encounter this protein directly after it enters the cell, which may subsequently result in cisplatin sequestration in ATP7A-expressing vesicles and therefore prevent its binding to nuclear DNA. This is supported by the previous findings that cellular accumulation of cisplatin is 2.5 times lower in the A2780cis cell line compared to the A2780 cell line, while DNA platination is, on average, reduced 5.4-fold [20]. It should be noted, however, that other factors such as increased glutathione levels in the A2780cis cell line (Zisowsky *et al.*, unpublished data) are also involved in regulating intracellular transport of cisplatin and cisplatin sensitivity of A2780cis cells.

## Conclusion

Taken together, our results suggest that changes in sub cellular localisation of copper efflux transporters may account for acquired resistance to cisplatin. Altered localisation of ATP7A and ATP7B in A2780cis cells is likely to result from a general defect in intracellular protein recycling, which may be related to the reduced lysosomal compartment in this cell line. Earlier studies have indicated that accumulation of cisplatin in the secretory pathway may require lysosomal function. Abnormalities in structure and function of the lysosomal compartment may promote sequestration of cisplatin away from its pharmacological target, nuclear DNA, due to incorrect localisation of transport proteins.

The results of the present study further indicate that sub cellular localisation of transport proteins can serve as a marker for detection of clinically relevant cisplatin resistance. Using modern imaging technology it should be possible to identify resistant tumours at an early stage of chemotherapy and to adjust the treatment accordingly. In order to validate this approach, further studies should focus on the transporter localisation in tumour cells isolated from different patients and correlation of protein localisation patterns with clinical response to cisplatin chemotherapy. The results may contribute to the development of new individualised therapeutic strategies in cisplatin treatment of ovarian carcinoma.

## Competing interests

The authors declare that they have no competing interests.

## Authors' contributions

CHW carried out cytotoxicity tests and some immunohistochemical staining experiments, IB performed some immunohistochemical staining experiments, GVK designed the study, performed the rest of the experiments and wrote the manuscript. UJ participated in the study design and data interpretation, and contributed to drafting the paper. JR participated in discussion of the results. All authors critically revised the manuscript, read and approved its final version.

## Pre-publication history

The pre-publication history for this paper can be accessed here:


